# Six decades of evidence: biological factors predominate among reported reasons for clinical drug failure: *A response to “Persistent clinical attrition does not establish animal models as a root cause of drug-development failures” by Peter MA Groenen*

**DOI:** 10.1016/j.namjnl.2026.100136

**Published:** 2026-09-23

**Authors:** Dilyana Filipova, Leyla Fox, Matthias Gossmann, Kathrin Herrmann, Andrew Knight, Bettina Lickiss, Christian Maass, Gaby Neumann, Merel Ritskes-Hoitinga, Tamara Zietek

**Affiliations:** a Doctors Against Animal Experiments (DAAE), Lustheide 85, 51427 Bergisch Gladbach, Germany; b European Coalition to End Animal Experiments (ECEAE), Lustheide 85, 51427 Bergisch Gladbach, Germany; c innoVitro GmbH, Artilleriestr. 2, 52428 Jülich Jülich, Germany; d Center for Alternatives to Animal Testing (CAAT), Johns Hopkins Bloomberg School of Public Health, 615 N. Wolfe Street, Baltimore, MD 21205, USA; e School of Veterinary Medicine, College of Environmental and Life Sciences, Murdoch University, Perth, 90 South St, Murdoch WA 6150, Australia; f School of Environment and Science, Griffith University, Brisbane, 170 Kessels Road, Nathan QLD 4111, Australia; g Faculty of Health and Wellbeing, University of Winchester, Sparkford Road, Winchester, Hampshire, SO22 4NR, UK; h ESQlabs GmbH, MPSlabs, 26683 Saterland, Germany; i Faculty of Veterinary Medicine, Utrecht University, Heidelberglaan 8, 3584 CS Utrecht, Netherlands; j Aarhus University, Nordre Ringgade 1, 8000 Aarhus C, Denmark

Dear Editor,

In our study, *Need for NAMs: A systematic evidence synthesis revealing over half a century of drug development failure*, we report that clinical drug attrition remained between approximately 80% and 91% across six decades (1960s–2010s), and that biological factors – including safety, efficacy, and pharmacokinetics (PK) – accounted for approximately 77% of reported clinical failures (Filipova et al., 2026). Dr. Groenen's letter raises several questions about our findings and their interpretation, which we address below.

## Clarifying what our paper claims and does not claim

Our two research questions were descriptive in scope. RQ1 maps trends in clinical drug attrition; RQ2 analyses the proportion of reported clinical failures attributable to biological factors – like safety, efficacy, and PK – versus non-biological factors such as commercial or strategic decisions. We did not measure the concordance or predictive validity of any specific preclinical method, including animal experiments, against clinical outcomes. Rather, we discussed the established practice of assessing these biological properties in animals during late-stage preclinical evaluation alongside the independently measured finding that they accounted for approximately 77% of reported clinical failures. This contextual relationship was discussed without treating it as a paired concordance analysis.

Although our analysis was not designed to quantify the proportion of failures specifically attributable to interspecies differences, this limitation does not make such differences irrelevant. Species-specific differences in physiology, disease mechanisms, PK, and toxicological responses are independently documented, including in studies cited by Dr. Groenen (Olson et al., 2000, Giblin et al., 2020). These differences therefore remain plausible and potentially important contributors to clinical attrition.

The letter suggests that our study identifies animal models as the "root cause" or "primary driver" of drug attrition. Neither claim appears in our study, nor do we claim singular causation. Although our study uses the term "primary driver" once, it refers to biological factors, not to animal models specifically. We therefore clarify this distinction to avoid attributing to our study a claim it does not make.

The letter also raises a concern about potential author bias despite the safeguards described in our publication. We take the concern about potential bias seriously. However, the letter does not identify a specific deviation in data extraction, study selection, protocol adherence, or the safeguards described in our publication. The disagreement therefore appears to concern interpretation rather than a demonstrated methodological problem.

## RQ2 concerns reported reasons for failure

Dr. Groenen writes that "Research Question 2 has therefore not been adequately answered". We interpret the scope of RQ2 differently. RQ2 asks what percentage of drug candidates fail for biological reasons versus non-biological reasons, as reported by the authors. Our synthesis answers this directly: across 36 datasets spanning 1963–2016, biological reasons accounted for approximately 77% of reported clinical failures on average.

The letter characterizes this as a confusion between "the location of failure (a biological endpoint)" and "its cause". Our analysis, however, extracted reasons for program discontinuation as reported by the primary studies, including inadequate efficacy, toxicity, and poor PK. It did not treat endpoints such as liver-enzyme levels or tumour size as reasons for failure. Nor did it seek to identify the ultimate causal mechanism of each failure or quantify the contribution of animal-model limitations. The 77% figure should therefore be understood as a synthesis of reported reasons. Considered alongside evidence on interspecies differences and variable animal-to-human concordance, this pattern warrants continued critical evaluation of the prevailing preclinical paradigm.

## Interpreting efficacy-related failure

Clinical-development decisions – including indication selection, endpoints, patient populations, and dosing – may contribute to efficacy-related failure. Dr. Groenen attributes inadequate efficacy largely to such decisions, including inadequate target validation and incomplete understanding of human pathophysiology. Hwang et al. (2016) report the distribution of failure categories (57% efficacy, 17% safety, 22% commercial), while Fogel (2018) and Harrison (2016) review factors associated with trial failure; these sources do not establish that efficacy failure is predominantly due to human decisions rather than species differences (Hwang et al., 2016, Fogel, 2018, Harrison, 2016). Our findings therefore do not exclude the contribution of development decisions, but the cited evidence does not establish them as the predominant explanation.

The letter further asserts that human-based NAMs “do not, in themselves, resolve” these knowledge gaps because they "inherit the same uncertainty about which target and which mechanism matter in human disease". The letter does not provide specific evidence to substantiate this claim. Human-derived NAMs can contribute to target and mechanism validation precisely because they operate in human biological contexts, reducing species-specific confounders (Song et al., 2026, Loewa et al., 2023, Wu et al., 2026, Sewell et al., 2026 29). Animal models are also routinely used for these purposes, despite repeated criticism of their limited translation to humans (Hartung, 2024 Apr 8, Pistollato et al., 2020 Jul, Seok et al., 2013 Feb 26, Marshall et al., 2023). These considerations suggest that efficacy failure should not be attributed exclusively or predominantly to human decisions without also evaluating the contribution of limitations in animal-based approaches.

## The preclinical survivor limitation

Preclinical failures were absent from our dataset. This limits assessments of model predictive validity but does not invalidate RQ1 or RQ2, which concern clinical attrition and the reported reasons for clinical failure, respectively. The absence of compounds discontinued during preclinical development also does not establish that animal studies correctly distinguished compounds unlikely to succeed in humans from those that might have succeeded, because the human outcomes of those compounds remain unknown. Notably, several concordance studies cited by Dr. Groenen acknowledge the same limitation in their own datasets (Olson et al., 2000, Giblin et al., 2020, Clark and Steger-Hartmann, 2018).

## Animal-to-human translation is context-dependent

The concordance literature supports a qualified, context-dependent interpretation rather than a general conclusion of reliable animal-to-human translation. For instance, Olson et al. reported concordance above 80% for selected effects, but overall concordance was lower at 71%, with concordance of 63% for non-rodents and 43% for rodents across human toxicities (Olson et al., 2000).

Similarly, the IQ Consortium analysis reported sensitivity of 48% and positive predictive value of 43%, sharply contrasting with specificity of 84% and negative predictive value of 86% (Monticello et al., 2017). These metrics answer different predictive questions but together illustrate that performance varies across endpoints and types of prediction.

Other studies have reported more limited concordance, including van Meer et al. (2012), who found that only 19% of serious post-marketing adverse reactions had been identified in animal studies, and Atkins et al. (2020), who identified limited concordance between animal and human toxicities in Phase I oncology trials (van Meer et al., 2012, Atkins et al., 2020). These studies concern specific populations, which limits their generalizability but does not negate their findings. Comparable qualifications apply to evidence of high concordance obtained from selected endpoints or study populations (Olson et al., 2000 1, Giblin et al., 2020, Clark and Steger-Hartmann, 2018, Monticello et al., 2017, Liu and Fan, 2026, Ineichen et al., 2024, Perel et al., 2007). In addition, the concordance literature is affected by the exclusion of compounds that did not progress to human testing, whose clinical outcomes remain unknown. In an attempt to find predictive success factors for animal to human translation, a scoping review of reported translational percentages was performed by Leenaars et al. (2019): translational percentages ranged from 0 to 100% and did not change over time (Leenaars et al., 2019). It was not possible to identify factors that would lead to successful translation from animals to humans.

Finally, the letter cites an umbrella review reporting that approximately half of animal-tested interventions reached human study, while approximately 5% ultimately obtained regulatory approval (Ineichen et al., 2024). Progression to human study is therefore not equivalent to clinical or regulatory success.

Taken together, the available evidence indicates that animal-to-human concordance varies by endpoint, species, study population, and development stage. The favourable estimates cited in the letter represent important findings, but they need to be considered alongside lower estimates and methodological limitations reported elsewhere in the literature. This broader evidence base supports continued evaluation of the predictive validity and human relevance of preclinical methods, rather than a general conclusion that animal studies reliably predict later clinical outcomes.

## The "economic equilibrium" interpretation

Dr. Groenen proposes that persistent attrition reflects an "economic equilibrium," shaped by fail-fast strategies, rising regulatory standards, and increasingly difficult biology. These factors may contribute to attrition, and our paper does not claim otherwise. However, the sources cited address declining R&D efficiency rather than quantifying the relative contributions of economic and biological factors to clinical attrition (Paul et al., 2010, Scannell et al., 2012). Across all 36 datasets in our RQ2 synthesis, biological reasons exceeded non-biological reasons, accounting on average for approximately 77% and 23.5% of reported failures, respectively. An "economic equilibrium" may influence portfolio decisions and timing of discontinuation, but it does not by itself account for the repeatedly reported predominance of safety, efficacy, and PK-related failure.

Kola and Landis reported a decline in PK-related attrition from approximately 40% in 1991 to below 10% in 2000, which Dr. Groenen interprets as redistribution rather than reduction of failure (Kola and Landis, 2004). The authors present redistribution as a possibility, not as a demonstrated conclusion, and also discuss limitations in the predictive value of animal models in several disease areas. Their findings do not determine whether improved predictive methods, including NAMs, can reduce attrition. Our paper makes no claim that any single methodological change will do so. It calls for systematic evaluation and modernization of preclinical approaches, alongside other improvements, to identify measures that genuinely improve translation.

## The decision-cascade interpretation and the supporting evidence

Dr. Groenen proposes that the predictive validity of the whole decision cascade – including target and indication choice, biomarkers, trial design, and "where they help, better human-relevant models" – better explains persistent failure than attribution to animal testing alone. We agree that decision quality influences attrition and that multiple factors contribute to clinical failure.

The AstraZeneca 5R analysis illustrates the potential value of improving several parts of the development process, reporting an increase in success rates from 4% to 19% (Morgan et al., 2018, Cook et al., 2014). However, the cited publications describe broader changes in target validation, PK modelling, biomarkers, patient selection, safety assessment, and the integration of human-relevant systems, including organoids. The analysis therefore does not isolate the contribution of animal-based testing or establish that reliance on animal models has remained unchanged. Notably, the original 5R analysis also reported that safety issues accounted for more than half of terminated projects (Cook et al., 2014).

Our paper similarly supports the need to evaluate the entire preclinical decision process. It additionally emphasizes the systematic evaluation and modernization of preclinical approaches, including NAMs, assessed against high, evidence-based standards and defined contexts of use – standards not historically applied consistently to animal models themselves (Knight, 2007, Varga et al., 2010, Green, 2015). This inconsistency can limit the reliability and interpretability of conclusions drawn from animal studies.

## Conclusion

Our systematic evidence synthesis shows that clinical failure rates remained between approximately 80% and 91% since the 1960s, peaking at approximately 91% in the 2000s–2010s. Biological factors accounted for approximately 77% of reported clinical failures on average and were therefore the predominant reported reasons for failure in the analysed datasets.

Dr. Groenen’s letter offers alternative interpretations of these findings. The cited evidence does not, however, establish that efficacy failures are predominantly attributable to clinical-development decisions, that animal-to-human translation is broadly reliable, or that attrition is primarily an economic equilibrium. Our paper does not propose a single solution or a monocausal explanation. It calls for systematic evaluation and modernization of preclinical approaches – including NAMs held to high, evidence-based, context-of-use standards. This is consistent with the letter’s own emphasis on a broader decision system, and with regulatory initiatives that increasingly encourage human-relevant methods. Notably, such explicit standardized predictive-validity standards have not historically been applied consistently across preclinical methodologies, including animal models, despite the longstanding use of animals in preclinical drug assessment. Continued, systematic monitoring of new methodologies and policy changes will be essential to identify evidence-based strategies that genuinely improve the translation of preclinical findings into successful therapies for patients.

Sincerely,

Dilyana Filipova and Tamara Zietek

On behalf of the responding authors

## CRediT authorship contribution statement

**Dilyana Filipova:** Writing – review & editing, Writing – original draft, Conceptualization. **Leyla Fox:** Writing – review & editing. **Matthias Gossmann:** Writing – review & editing. **Kathrin Herrmann:** Writing – review & editing, Writing – original draft. **Andrew Knight:** Writing – review & editing, Writing – original draft. **Bettina Lickiss:** Writing – review & editing. **Christian Maass:** Writing – review & editing. **Gaby Neumann:** Writing – review & editing. **Merel Ritskes-Hoitinga:** Writing – review & editing. **Tamara Zietek:** Writing – review & editing, Writing – original draft, Conceptualization.

## Declaration of competing interest

The authors declare the following financial interests/personal relationships which may be considered as potential competing interests:Bettina Lickiss reports a relationship with innoVitro GmbH that includes: employment. Matthias Gossmann reports a relationship with innoVitro GmbH that includes: employment. Christian Maass reports a relationship with ESQlabs GmbH that includes: employment. Given her role as a member of the Editorial Board of the NAM Journal, Merel Ritskes-Hoitinga had no involvement in the peer review of this article and had no access to information regarding its peer review. Full responsibility for the editorial process for this article was delegated to another journal editor. If there are other authors, they declare that they have no known competing financial interests or personal relationships that could have appeared to influence the work reported in this paper.

## Data Availability

No data was used for the research described in the article.
